# In situ characterization of protein aggregates in human tissues affected by light chain amyloidosis: a FTIR microspectroscopy study

**DOI:** 10.1038/srep29096

**Published:** 2016-07-04

**Authors:** Diletta Ami, Francesca Lavatelli, Paola Rognoni, Giovanni Palladini, Sara Raimondi, Sofia Giorgetti, Luca Monti, Silvia Maria Doglia, Antonino Natalello, Giampaolo Merlini

**Affiliations:** 1 Department of Biotechnology and Biosciences, University of Milano-Bicocca, Piazza della Scienza 2, 20126 Milano, Italy; 2 Department of Physics, University of Milano-Bicocca, Piazza della Scienza 3, 20126 Milano, Italy; 3 Amyloidosis Research and Treatment Center, Foundation IRCCS Policlinico San Matteo, and Department of Molecular Medicine, University of Pavia, Viale Golgi 19, 27100 Pavia, Italy; 4 Department of Molecular Medicine, Institute of Biochemistry, University of Pavia, via Taramelli 3b, 27100 Pavia, Italy

## Abstract

Light chain (AL) amyloidosis, caused by deposition of amyloidogenic immunoglobulin light chains (LCs), is the most common systemic form in industrialized countries. Still open questions, and premises for developing targeted therapies, concern the mechanisms of amyloid formation *in vivo* and the bases of organ targeting and dysfunction. Investigating amyloid material in its natural environment is crucial to obtain new insights on the molecular features of fibrillar deposits at individual level. To this aim, we used Fourier transform infrared (FTIR) microspectroscopy for studying *in situ* unfixed tissues (heart and subcutaneous abdominal fat) from patients affected by AL amyloidosis. We compared the infrared response of affected tissues with that of *ex vivo* and *in vitro* fibrils obtained from the pathogenic LC derived from one patient, as well as with that of non amyloid-affected tissues. We demonstrated that the IR marker band of intermolecular β-sheets, typical of protein aggregates, can be detected *in situ* in LC amyloid-affected tissues, and that FTIR microspectroscopy allows exploring the inter- and intra-sample heterogeneity. We extended the infrared analysis to the characterization of other biomolecules embedded within the amyloid deposits, finding an IR pattern that discloses a possible role of lipids, collagen and glycosaminoglycans in amyloid deposition *in vivo*.

The conformational changes of specific proteins and peptides from their functional state into fibrillar aggregates are associated with an expanding number of human diseases with an overall high social impact. This class of conditions includes neurodegenerative disorders, such as Alzheimer’s disease, as well as localized and systemic amyloidoses. In the latter, in particular, extracellular amyloid deposition is widespread, occurring at sites distant from those of protein production, from blood-borne amyloidogenic precursors^1^,^2^,^3^,^4^. Under electron microscopy examination, the amyloid deposits are composed of 10 nm-wide extracellular unbranched fibrils, remarkably similar despite the nature of the amyloidogenic precursor^5^. Demonstrating the presence of amyloid deposits in tissues is the first necessary step for the diagnosis of amyloidoses; in the clinical setting, this is most commonly achieved through the use of histological dyes, such as Congo red, which display specific tinctorial and spectral properties when bound to the amyloid fibrils^6^.

Immunoglobulin light chain (AL) amyloidosis is the most common systemic form in industrialized countries and is caused by misfolding-prone amyloidogenic immunoglobulin light chains (LCs) secreted from a bone marrow plasma cell clone^7^,^8^. The clinical presentation of AL amyloidosis is heterogeneous, and depends on which sites are target of amyloid deposition. With the exception of the central nervous system, virtually every organ can be involved in this form, including heart, kidneys, liver, autonomic and peripheral nervous system, gastrointestinal tract, soft tissues^7^. Subcutaneous abdominal fat is also a frequent site of deposition and, due to the easiness of acquisition, is the preferred biopsy tissue in suspected patients^9^.

Despite important advancements in research, many key questions are still open in the field of systemic amyloidoses, from the mechanisms leading to misfolding and aggregation, to the bases of organ targeting and dysfunction. Response to these questions would be a premise for developing novel therapies aimed at interfering with specific components of the pathogenic cascade.

Investigating how amyloid fibrils form in the biological environment, in particular, is a major critical point. For some proteins (as the highly amyloidogenic D76N mutant and ∆N6 truncated forms of β-2 microglobulin) the process of fibrillogenesis has been successfully reproduced *in vitro* under physiological conditions^10^. In the case of amyloidogenic LCs, instead, *in vitro* fibrillogenesis of the full-length precursors is typically achievable only under strong destabilizing conditions, such as high temperature, extreme pH, and/or long incubation times^11^,^12^,^13^. Moreover, studies *in vitro* cannot comprehensively take into account the complete ensemble of biochemical and biophysical factors, including the components of the environment, in which amyloidogenesis takes place. The characterization of amyloid aggregates in their natural environment – *i.e.* within patients’ tissues – is therefore an important open issue, and the most reliable mean to obtain biologically relevant information. In this view, the use of biophysical approaches allowing amyloid analysis *in situ* would offer the opportunity to investigate the aggregates exactly where amyloid deposition takes place, and to gain information on the molecular features of deposits at the individual level.

Fourier transform infrared (FTIR) microspectroscopy is a powerful technique that enables to obtain information on the composition and structures not only of isolated biomolecules^14^,^15^,^16^, but also of complex biological systems, such as intact cells and tissues^17^,^18^,^19^,^20^. This label-free spectroscopic approach has been successfully applied to the characterization of tissues affected by amyloid deposition in neurodegenerative diseases, allowing to detect the intermolecular β-sheet structures *in situ*, and also to provide a molecular fingerprint of other biomolecules within or surrounding the amyloid aggregates^19^,^20^,^21^,^22^,^23^,^24^. The FTIR study of tissues affected by systemic or localized amyloidoses, however, has not been described yet.

In this work, we have employed FTIR microspectroscopy to analyze unfixed human tissues from patients affected by systemic light chain amyloidosis. Notably, the use of an infrared microscope made it possible to measure the IR response from selected and spatially distinct areas of the sample, enabling the investigation of its heterogeneity and therefore to explore the colocalization of amyloid deposits with other biomolecules.

We show that the usefulness of FTIR microspectroscopy, in this setting, is twofold. Firstly, in fact, the detection of the *in situ* marker band of the aggregates allowed demonstrating the presence of amyloid deposits. In second instance, the possibility to measure unfixed tissue sections^25^ allowed to detect important peculiarities in the spectral features of other biomolecules in areas enriched with aggregates, suggesting a role in particular of lipids in amyloid deposition *in vivo*. This study indicates that FTIR microspectroscopy analysis of amyloid-affected tissues is a possible tool for the diagnosis of amyloidosis, and paves the way for further investigations aimed to the *in situ* characterization of fibrillar deposits for basic research as well as for clinical purposes.

## Results

### Clinical and histological features of AL amyloid-infiltrated human tissues

In order to characterize the amyloid deposits within tissues, we measured the infrared absorption of amyloid-infiltrated human samples (ventricular myocardium and subcutaneous abdominal fat) from patients affected by systemic light chain (AL) amyloidosis. Amyloid-negative adipose and heart tissue samples were used as controls. The main clinical and histological features of samples are reported in Table 1.

**Figure 1:**
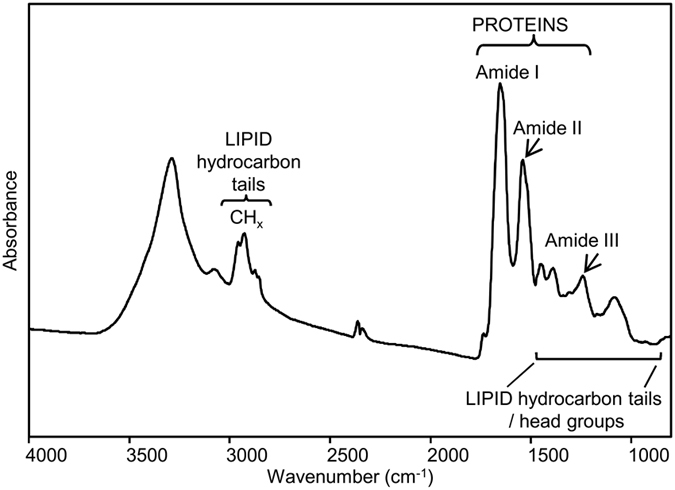
The FTIR absorption spectrum of human cardiac tissue. A representative IR spectrum of the cardiac sample HT1 from an area of ~40 μm × 40 μm is shown in the mid IR range. The assignment of selected bands to the tissue biomolecules is reported.

A representative IR spectrum of an area of the cardiac tissue sample HT1 is reported in Fig. 1, with the assignment of selected bands to the main biomolecules of the tissue. This spectrum is exemplificative of a possible outcome of the FTIR analysis of this tissue, and of the potential information that can be obtained from specific spectral regions.

In particular, between 1700 and 1500 cm^−1^, the spectrum is dominated by the protein amide I and amide II bands, respectively due mainly to the C = O stretching and the NH bending of the peptide bond. The amide I band is particularly sensitive to the protein secondary structures and to the presence of intermolecular β-sheets in protein aggregates^14^,^26^,^27^,^28^. Furthermore, the spectral range between 3000 and 2800 cm^−1^ is dominated by the lipid hydrocarbon tail absorption, that is also present between 1500 and 1200 cm^−1^. In this last spectral region the absorption of the lipid head groups, that of the protein Amide III, and of the phosphates also occurs^14^,^16^,^29^.

The detailed analysis of these spectral regions in the analyzed tissues (Table 1) is reported in the following sections, to explore the potential of FTIR microspectroscopy for the *in situ* molecular characterization of amyloid deposits.

### FTIR investigation of the structural properties of amyloid positive cardiac tissues: Amide I band analysis

We investigated the infrared response of cardiac tissues in the Amide I band to get insights into the protein structural properties, and in particular to disclose marker bands of amyloid deposits.

**Figure 2:**
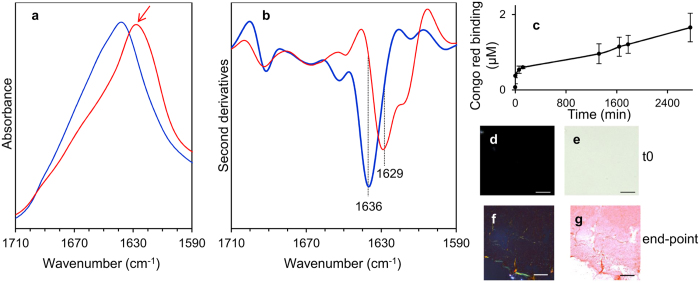
Amide I band components of recombinant native and fibrillar LC. (**a**) FTIR absorption spectra of native λ LC (blue line) and of fibrils obtained *in vitro* from the same protein (red line) whose sequence was derived from the protein produced by the patient who provided the HT3 sample. The red arrow indicates the marker band of protein aggregates around 1629 cm^−1^. (**b**) Second derivatives of the spectra reported in A. (**c**) Time course of the *in vitro* fibrillogenesis of amyloidogenic isolated LC HT3 followed by Congo red binding. Data are expressed as the mean ± standard deviation, n = 4. (**d,e**) 50 μL aliquot of time 0 fibrillogenesis were stained with Congo red dye and visualized under polarized (**d**) and light (**e**) microscopy, showing the typical apple green birefringence of amyloid fibers under polarized light. Magnification 200×, scale bar: 100 μm. (**f,g**) as in (**d,e**), but of end-point fibrillogenesis.

To obtain the reference IR responses of fibrillar and non-fibrillar LCs, we measured firstly the FTIR absorption spectra (Fig. 2a) from the native and from the fibrillar recombinant full-length LC, whose sequence corresponds to that produced by the patient who provided the HT3 sample^12^.

To better resolve the overlapping components in the measured spectra, we performed the second derivative analysis, as shown in Fig. 2b^30^. In particular, the second derivative spectrum of the purified native LC protein displayed a main peak at ~1636 cm^−1^, along with the peak at ~1691 cm^−1^, which are unambiguously assigned to the intra-molecular β-sheet structures of the native protein^14^. The minor component at ~1652 cm^−1^ occurred in the spectral region of α-helix and random coil absorption^14^. Noteworthy, compared to the native protein, the β-sheet peak of *in vitro* fibrils (Fig. 2b) was found to be shifted down to ~1629 cm^−1^, indicating the presence of intermolecular β-sheet structures, as expected^26^,^27^,^28^, and as confirmed by the Congo red analysis (Fig. 2c,f,g). For comparison, we reported also the Congo red staining of time 0 of the *in vitro* fibrillogenesis (Fig. 2d,e).

**Figure 3:**
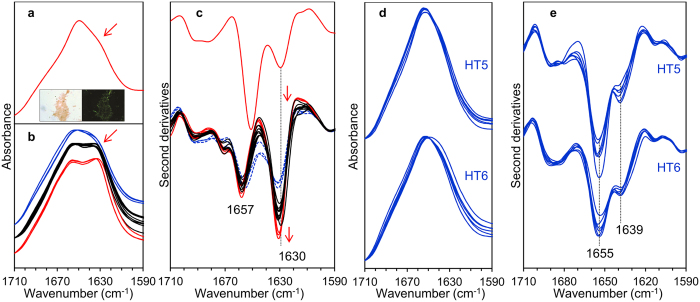
Amide I band components in cardiac tissues. (**a**) FTIR absorption spectrum of water-extracted fibrillar material from the HT1 tissue. Inset: Congo red staining visualized under light (left) and polarized (right) microscopy. Magnification 200×. (**b**) Representative absorption spectra of different areas within the HT1 tissue, characterized by different extents of protein aggregates (increasing from top to bottom). (**c**) Second derivatives of the spectra reported in (**a**) (upper spectrum) and in (**b**) (bottom spectra). The red arrows indicate the marker band of protein aggregates around 1630 cm^−1^. (**d**) FTIR absorption spectra of different areas within the amyloid negative tissues HT5 and HT6. (**e**) Second derivatives of the spectra reported in (**d**).

We then measured the *ex vivo* amyloid fibrils isolated from the heart of patient HT1 (Fig. 3a) and we compared their IR response with that obtained from different areas of the cardiac tissue from the same patient (Fig. 3b). The second derivative spectra of extracted amyloid materials (see Fig. 3c, for a representative spectrum) are characterized by a main peak at 1630–1626 cm^−1^, due to intermolecular β-sheet structures, in agreement with the results obtained from the fibrils of the recombinant protein (Fig. 2) and with Congo red analysis (see inset of Fig. 3a). The presence of a component at 1650–1645 cm^−1^ points out that the extracted aggregates also contained polypeptides in random coil and α-helix secondary structures^14^.

As shown in Fig. 3c, the tissue spectra displayed a component at ~1657 cm^−1^ due to α-helix/random coil structures of the whole protein content, and a second component at ~1630 cm^−1^, which occurred in the same spectral region of the intermolecular β-sheet response detected in the *in vitro* and *ex vivo* fibrils. Therefore, the ~1630 cm^−1^ band can be considered a marker of amyloid aggregates in tissues. The assignment of this band to protein aggregates is also supported by the analysis of the Amide II band (Supplemental Figure S1), which could reflect variations of the protein secondary structures and aggregation^31^,^32^. As shown in Supplemental Figure S1, the appearance and increase of the ~1630 cm^−1^ band occurred simultaneously to the decrease in the intensity of the Amide II component at ~1545 cm^−1^ and to the appearance/increase of a shoulder around 1531 cm^−1^, overall spectral features already reported for thermal induced aggregates^33^.

In most of the HT1 tissue spectra, the intensity of the ~1630 cm^−1^ peak was higher than that due to the α-helices/random coils (Fig. 3c), indicating that this sample was particularly enriched in amyloid deposits. However, the different intensity of this marker band among spectra from different areas testifies the heterogeneity of the sample. This is not surprising, since amyloid-positive samples are often characterized by a non-uniform distribution of the deposits^34^.

Noteworthy, the ~1630 cm^−1^ amyloid-marker band was not observed in any of the analyzed areas of the two amyloid-negative heart samples, as shown in Fig. 3d,e. Particularly, in the negative controls the β-sheet peak was detected at ~1639 cm^−1^, mainly due to intramolecular native β-sheets^26^,^28^,^32^. This result has been also confirmed by the Amide II band analysis (see Figure S1), whose spectral features are similar to those found for the amyloid-free areas of amyloid positive tissues, as well as by the Congo red staining of extracted materials from the heart amyloid negative tissue HT6 (Figure S2).

### FTIR analysis of the main biomolecules embedded in amyloid deposits in cardiac tissues

**Figure 4:**
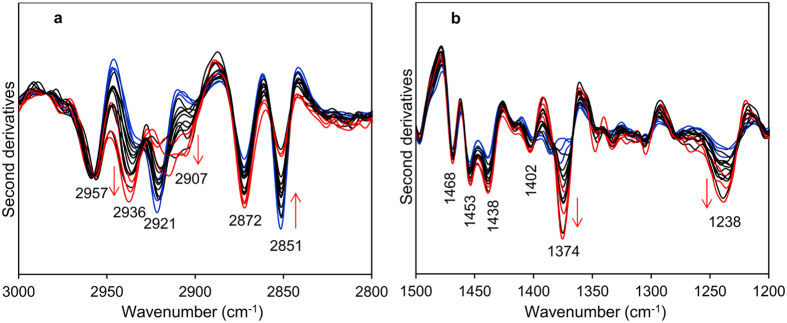
Analysis of the FTIR absorption in the 3000–2800 cm^−1^ and 1500–1200 cm^−1^ spectral ranges of the cardiac tissue HT1 positive for AL λ amyloidosis. (**a**) FTIR second derivative analysis of the lipid hydrocarbon tail absorption in the stretching region of the same sample areas reported in Fig. 3b. The red arrows point to the spectral changes mainly associated to cholesterol. (**b**) Second derivatives of the same spectra shown in (**a**). The peak position of lipid hydrocarbon tail deformation and head groups are indicated. In particular, the red arrows point to the increase of the cholesterol/GAG absorption at ~1374 cm^−1^ and to the Amide III/GAGs/phosphates at ~1238 cm^−1^.

We then further analyzed the spectra, to investigate possible variations of the lipid content among areas characterized by different enrichment in amyloid aggregates. To this aim, we focused the FTIR analysis between 3000–2800 cm^−1^, a spectral range dominated by the lipid hydrocarbon tail response (Fig. 4a). As shown, the infrared absorption was found to be complex: in fact, besides the stretching modes due to the CH_3_ (~2957 cm^−1^ and 2872 cm^−1^) and CH_2_ (~2921 cm^−1^ and 2851 cm^−1^) groups mainly of fatty acid acyl chains^16^,^29^, other two absorptions have been detected at ~2936 cm^−1^ and at ~2907 cm^−1^, which can be assigned mainly to the CH_2_ vibrations that characterize the spectrum of cholesterol^35^,^36^,^37^. Noteworthy, we observed that, within the same tissue, the spectra with higher intermolecular β-sheet content (see red lines in Fig. 3b,c, bottom spectra) displayed also a higher intensity of these two absorptions and a lower intensity of the two CH_2_ bands at ~2921 cm^−1^ and 2851 cm^−1^ (see red lines Fig. 4a). Interestingly, this spectral behavior reflects a rearrangement of the lipid composition and structure in tissue areas enriched in amyloid deposits.

To better investigate the lipid response, we extended the analysis to the spectral range between 1500–1200 cm^−1^, where the deformation modes of the hydrocarbon tail CH_3_ and CH_2_ groups, as well as the absorption of lipid head groups mainly occur (Fig. 4b). In this spectral region, in particular, the second derivative spectra are characterized by a number of well resolved bands at ~1468 cm^−1^, due to the overlapping absorption of CH_2_ and CH_3_, and at ~1453 cm^−1^, 1438 cm^−1^ and 1374 cm^−1^, due to CH_3_^16^,^29^,^38^. In addition, the ~1374 cm^−1^ component can also be due to the absorption of acetyl amino group of glycosaminoglycans (GAGs)^39^, one of the main components of the extracellular matrix. Moreover, the ~1402 cm^−1^ band can be mainly assigned to the CH_3_ bending vibration of the N(CH_3_)_3_, which is the head group of phosphatidylcholine (PC)^16^. Finally, a broad and complex absorption is detected between 1260–1220 cm^−1^, due to the overlapping contributions of phosphate groups mainly from phospholipids, of the sulfate group of GAGs, and of the protein Amide III band^14^,^16^,^39^. In this regard, it is also known that collagen, a protein abundant in the extracellular matrix, displays a high absorption around 1240 cm^−1 ^40,41. Noteworthy, it has been reported that the ~1230 cm^−1^ peak of the phosphate groups is upshifted in the presence of cholesterol^42^. Interestingly, we found that, among the spectra acquired from HT1 sample, those characterized by a higher intensity of the aggregate marker band (Fig. 3b, red lines) displayed a higher intensity of the ~1374 cm^−1^ and of the ~1238 cm^−1^ bands. In addition, the simultaneous increase in intensity of the ~2936 cm^−1^, 2907 cm^−1^ and 1374 cm^−1^ absorptions suggests an enrichment in cholesterol^36^,^43^, in the same tissue areas affected by amyloid deposits (Fig. 4b). This result is also supported by the upshift of the complex absorption between 1260–1220 cm^−1^, from ~1233 cm^−1^ to ~1238 cm^−1^ (see also Figure S3)^42^, which is associated to the increase of the intermolecular β-sheet response. All this considered, the increase in intensity of the ~1238 cm^−1^ absorption suggests a colocalization of collagen, GAGs and amyloid aggregates.

### FTIR analysis of cardiac tissues positive to AL extended to different patients

To assess if the spectral features observed for the HT1 sample are common to cardiac tissues positive for AL, we performed the same analysis on samples from three additional patients.

**Figure 5:**
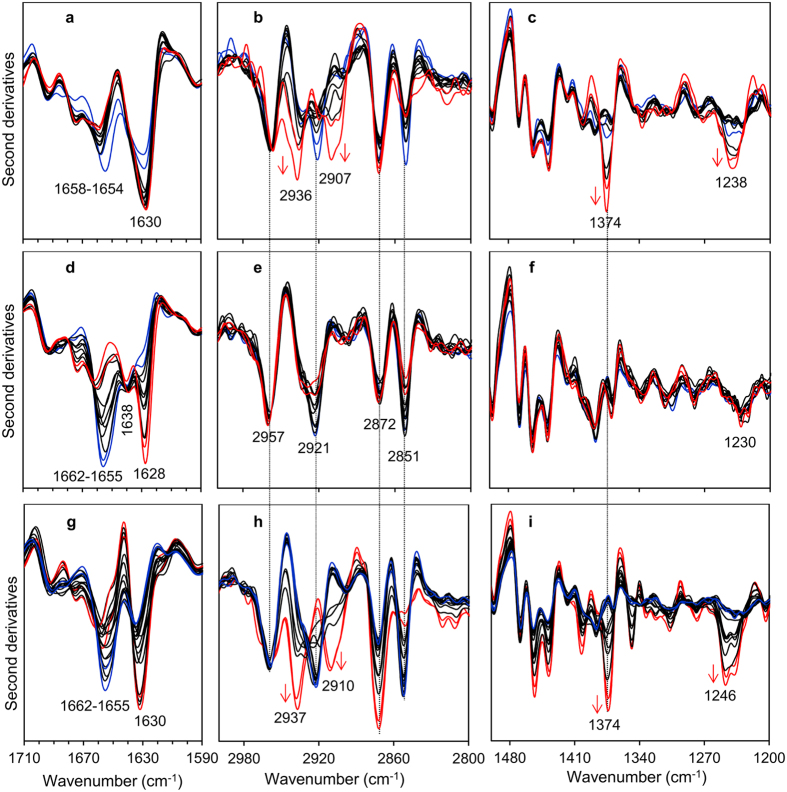
IR spectral features of HT2, HT3 and HT4 cardiac tissues positive to AL amyloidosis. Amide I band analysis of HT2 (**a**), HT3 (**d**) and HT4 (**g**) cardiac tissues. Cholesterol/GAG/phosphate analysis of HT2 (**b,c**), HT3 (**e,f**) and HT4 (**h,i**) cardiac tissues. Representative second derivative spectra of areas characterized by different extents of protein aggregates are reported. The peak positions of the spectral components discussed in the text are indicated.

In Fig. 5a we reported the second derivative spectra in the Amide I band of the HT2 tissue. As it can be seen, the spectra displayed features similar to those observed for the HT1 sample (see Fig. 3c): a main peak at ~1630 cm^−1^, due to intermolecular β-sheets in the protein aggregates, and a component at 1658–1654 cm^−1^ due to α-helix and random coil structures^14^,^26^,^27^,^28^.

We then analyzed also the lipid spectral features (Fig. 5b,c). Interestingly, again we observed the same spectral behavior described for the HT1 tissue (see Fig. 4a,b). In particular, the spectra from areas of the tissue characterized by a higher amount of aggregates displayed also a higher absorption at ~2936 cm^−1^, 2907 cm^−1^, and 1374 cm^−1^ mainly due to cholesterol^36^,^43^ (Fig. 5a–c, red lines), compared to the spectra representing areas less enriched in amyloid deposits. Moreover, the absorption at ~1238 cm^−1^ was also found to increase in intensity in the sample areas with a higher extent of protein aggregates (Fig. 5a,c, red lines), again reflecting a possible colocalization of collagen, GAGs, and cholesterol with the amyloid deposits.

In Fig. 5d–f, we reported the IR response of cardiac tissue HT3, from the same patient from whom the sequence of the recombinant amyloidogenic LC analyzed in Fig. 2 derived. Analogously to what observed in the previous cases, we detected the IR component assigned to intermolecular β-sheet structures of protein aggregates, which was found at ~1628 cm^−1^ (Fig. 5d). In addition, the absorption observed at ~1638 cm^−1^ was assigned to residual intramolecular β-sheets^14^. Furthermore, the α-helix/random coil component was detected between 1662–1655 cm^−1^. As shown, significant heterogeneity among spectra acquired from distinct tissue areas was observed, indicating the presence of regions with a different content of amyloid aggregates.

Interestingly, the lipid profile was instead very different from that detected in HT1 and HT2 tissues. In particular, the bands at ~2936 cm^−1^, 2907 cm^−1^ and at ~1374 cm^−1^, at high intensity in amyloid enriched areas of HT1 and HT2 tissues, were not evident in HT3 sample (Fig. 5e,f), indicating a low or negligible cholesterol content. Moreover, the CH_2_ (~2921 and 2851 cm^−1^) and CH_3_ (~2957 and 2872 cm^−1^) bands -mainly due to acyl chain absorption- were found to be well resolved^16^. In addition, the absorption between 1260 cm^−1^ and 1220 cm^−1^, at lower intensity compared to the other samples, was downshifted to ~1230 cm^−1^, a peak position that can be mainly assigned to phosphates of phospholipids^16^. All this considered, these results suggest that the amyloid deposits of HT3 were not enriched neither in cholesterol, nor in collagen and GAGs.

Finally, the spectral behavior of sample HT4 (Fig. 5g–i) resembled that detected for the tissue HT1 (Figs 3 and 4). Particularly, the more intense the intermolecular β-sheet band around 1630 cm^−1^, indicating areas enriched in amyloid deposits, the more intense the components at ~1374 cm^−1^, 2937 and 2910, spectral features indicating the presence of GAGs and cholesterol. 

Overall, we did not observe significant differences in the spectral behavior of samples that could be related to their mode of acquisition, i.e. *ex vivo* versus from autopsy.

### Protein secondary structure analysis in subcutaneous abdominal adipose tissues

Subcutaneous periumbilical fat is the tissue of election for histological diagnosis of systemic amyloidosis in suspected patients. Therefore, we explored the possibility of detecting amyloid deposits by FTIR microspectroscopy in this tissue (Table 1). Moreover, in order to verify the specificity of the analysis, we compared the infrared response from patients affected by AL amyloidosis with the signal obtained from non-affected control fat.

**Figure 6:**
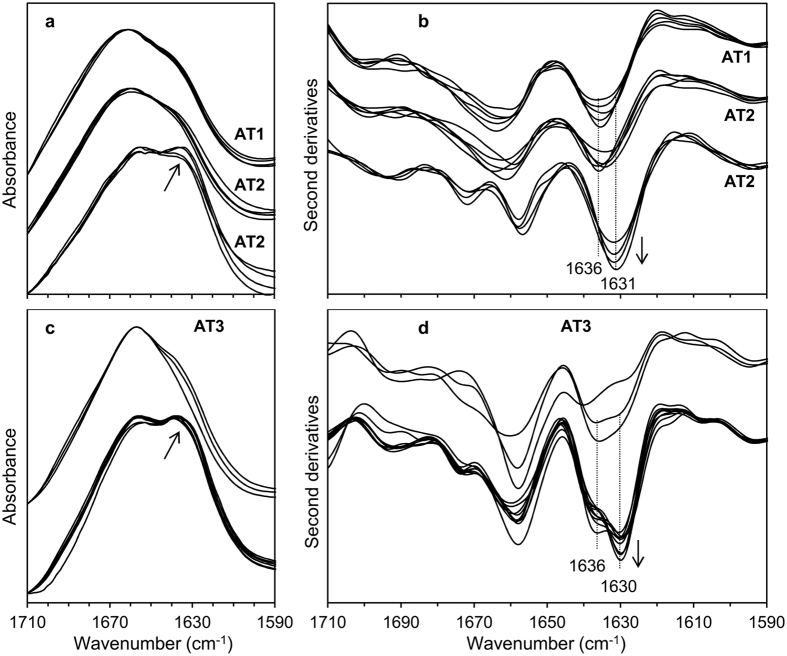
Amide I band analysis of the adipose tissues positive to AL amyloidosis. (**a**) FTIR absorption spectra from different areas of AT1 tissue (negative control) (upper spectra) and of AT2, positive for AL amyloidosis (middle and bottom). (**b**) Second derivative analysis of the spectra reported in (**a**). (**c**) FTIR absorption spectra of different areas of AT3 tissue, positive for AL amyloidosis, characterized by different extents of protein aggregates. (**d**) Second derivative analysis of the spectra reported in (**c**). The arrows indicate the aggregate marker band.

In Fig. 6a, we reported the Amide I absorption of areas in the negative adipose tissue sample AT1 (Fig. 6a, upper spectra) and in different areas of the AL-positive tissue AT2, enriched (bottom) or not enriched (middle) in amyloid aggregates. All the measured spectra are characterized by a band in the spectral range between 1664–1655 cm^−1^, due to α-helix and random coil structures of the total protein content^14^. In addition, AL-positive tissue areas are characterized by a band at ~1631 cm^−1^ (arrow in 6a), due to intermolecular β-sheets of protein aggregates^26^,^27^,^28^, absent in the negative control. Notably, in the patient’s tissue we also detected areas with the Amide I spectral features very similar to those observed for the negative control (Fig. 6a, middle spectra), indicating areas free of amyloid deposits. To obtain a better insight in the β-sheet response of the overall proteins, we analyzed the second derivative spectra (Fig. 6b).The negative control is characterized by a peak at ~1636 cm^−1^, assigned to intramolecular β-sheets^14^. The same spectral behavior characterized some spectra of the positive sample, confirming the presence of areas not containing amyloid deposits.

Importantly, other spectra from the AL positive sample displayed the component at ~1631 cm^−1^, assigned to intermolecular β-sheets, representative of areas enriched in light chain fibrils (see also the Amide II band analysis reported in Figure S4).

Similar results were obtained for the positive tissue AT3, as reported in Fig. 6c,d. Also in this case, we found areas characterized by an intense component at ~1630 cm^−1^, marker of the amyloid deposits, besides areas displaying a main IR absorption at ~1658 cm^−1^, due to α-helix and random coil structures, and a component at ~1636 cm^−1^ assigned to intramolecular β-sheets^14^,^26^,^28^.

As described for the cardiac tissues, we also analyzed the 3000–2800 cm^−1^ and 1500–1200 cm^−1^ spectral ranges to investigate the absorption of other biomolecules (data not shown). However, the very high intrinsic lipid content of the adipose tissue dominates these spectral ranges, covering possible specific spectral changes due to different biomolecules.

## Discussion

In this work, we characterized the *in situ* infrared response of amyloid deposits within cardiac and adipose human tissues from patients affected by systemic AL amyloidosis. This spectroscopic approach is label free and makes it possible to rapidly obtain a biochemical fingerprint of unfixed specimens, providing information on the content and on the structural properties of the main biomolecules in the tissue^17^,^19^,^24^. Notably, the use of an infrared microscope coupled to the FTIR spectrometer allowed collecting the infrared spectrum from selected sample areas of ~40 μm × 40 μm, enabling us to map the tissue response and to document the intra-sample heterogeneity, using minimal amounts of material.

Overall, our results demonstrate that FTIR tissue analysis has a twofold applicability in the context of systemic amyloid diseases: as a diagnostic instrument and as a tool to investigate the role of important biomolecules–including lipids, collagen and GAGs–in amyloid deposition *in vivo*.

The first application is related to the fact that, in all the positive samples analyzed, the specific spectral features of the amyloid aggregates were identified^26^,^27^,^28^. In particular, the absorption between 1631–1626 cm^−1^, pathognomonic of the purified fibrils and due to intermolecular β-sheets, can be considered as an *in situ* marker band of amyloid deposits; we should note that the peak position is indicative of the hydrogen bonding strength in the protein aggregates: stronger hydrogen bonds correspond to lower wavenumbers^26^. This result, therefore, indicates that FTIR microspectroscopy is an effective tool to detect the presence of fibrillar aggregates in clinical specimens and to obtain useful information on the compactness of the aggregate structures. Moreover, this approach is useful to investigate intra-sample heterogeneity; in fact, areas containing the amyloid-marker band in positive samples are clearly distinguishable from negative areas within the same samples, and from negative tissues. Furthermore, differences in the IR spectral behavior among patients can also be evaluated, as discussed later.

Importantly, in contrast with Congo red staining examination, the detection of the specific marker band is not operator-dependent, and is not affected by subjective interpretation of the pathologist^44^,^45^.

The potential of FTIR in clinical diagnostics for cell and tissue analysis is recognized, and this technique now holds the potential to complement modern imaging techniques in several areas of pathology^20^,^46^,^47^. Indeed, the features described in this study indicate that the FTIR identification of the intermolecular β-sheets marker band could be a very promising new tool for the diagnosis of amyloid diseases, whose performances in terms of sensitivity, specificity, and adaptability to specific samples as fine needle fat aspirates^45^ deserve to be investigated in clinical cohorts of patients. Furthermore, the possibility to monitor protein aggregation and disaggregation in fat pad is of great relevance in view of the novel anti-amyloid therapies based on passive immunization aiming at accelerating the removal of amyloid deposits^48^,^49^,^50^.

In addition to its diagnostic potential, FTIR analysis has shown its usefulness in providing information on other tissue molecules, allowing to study the features associated with amyloid deposits without perturbing the tissue structure. Indeed, the comprehensive FTIR description of the deposits *in situ* highlights the correlation between protein aggregation and the biological environment; the main considerations that can be made in this regard are presented below.

First, the second derivative analysis of the spectra, which allows discriminating between intra- and intermolecular β-sheets^14^,^26^,^28^, demonstrated that in some amyloid-positive areas the two β-sheet populations coexist. This suggests the absorption by other proteins embedded in or close to the amyloid deposits, which is in line with the well-known notion that specific proteins, likely in a non-fibrillar state, co-localize with fibrils^51^,^52^.

Moreover, in amyloid-positive areas, we also found a higher absorption of the complex band at ~1238 cm^−1^, tentatively assigned mainly to collagen and GAGs^39^,^40^,^41^. These molecules are major components of the extracellular matrix and have been shown to modulate amyloid formation from several proteins *in vitro*^53^,^54^,^55^. The increase in the ~1238 cm^−1^ band suggests that they share the same tissue volume as amyloid deposits, and is in line with the experimental notions that nascent collagen induces the growth of light chain fibrils^56^, and that GAGs tune LC aggregation, depending on the specific LC sequence, GAG type and sulfation^11^,^57^. In addition, a recent proteomic analysis of amyloid-positive human fat has shown an increase in collagen VI and heparan sulfate proteoglycans (HSPG) in affected samples^58^. The interest of the present observations stands in their ability to demonstrate the spatial correlation between these matrix components and fibrils *in vivo*, supporting the potential role of the former in the disease processes.

It is of interest, moreover, that sample areas enriched in amyloid aggregates display a peculiar lipid response in most of the analyzed heart samples. In particular, the higher is the protein aggregate component, the higher is the intensity of the bands at ~2936 cm^−1^, 2907 cm^−1^, and 1374 cm^−1^, whose simultaneous presence has been assigned mainly to cholesterol^36^,^43^.

This novel result is in agreement with what previously reported for systemic ATTR and dialysis-related amyloidoses, in which lipids and lipoproteins enriched in cholesterol were found to co-localize with amyloid deposits at immunohistochemistry, especially in the myocardium^59^. Co-localization of cholesterol and amyloid fibrils is also well documented in the case of Aβ amyloid plaques^60^,^61^, whereas, to our knowledge, this is the first report of the enrichment of this lipid in amyloid-positive regions in AL amyloidosis. Although these observations strongly suggest a pathophysiological role of cholesterol in protein misfolding diseases, the precise contribution of this and other lipids in amyloid pathology is still matter of study^62^,^63^,^64^. This aspect has been especially investigated in Alzheimer’s disease, in which generation and clearance of Aβ protein, and its interaction with cells and membranes, are influenced by this molecule^65^,^66^. Our results demonstrate a connection between lipid distribution and amyloidogenesis also in AL amyloidosis, further highlighting the presence of common features among different protein misfolding diseases.

As described in the “Results” section, indeed, heterogeneity was also observed among samples. For instance, the HT3 specimen was characterized by a lower intensity of absorption of the cholesterol components, and by a higher intra-sample heterogeneity, with numerous areas free of amyloid deposits. This behavior is not surprising, considering the high variability of presentation and clinical features of AL patients. This complexity may be at least partially related to the peculiar primary sequence of each monoclonal light chain, which translates into heterogeneous biophysical properties, organ tropism and toxicity of the soluble (mis)folded protein and of its amyloid aggregates^3^,^67^. In this regard, the described approach interestingly could be a useful method for disclosing intrinsic differences among patients’ samples, both in terms of aggregate structure and molecular composition, possibly providing novel information on the biological bases of AL amyloidosis.

Notably, considering the *in situ* intermolecular β-sheet response, both the intensity and the specific peak position of the IR marker band provide useful information on the aggregate core properties, including the strength of the hydrogen bonds^14^,^26^ that affects the aggregate compactness. Interestingly, an upshift of the α-helix band was also observed simultaneously to the enrichment in β-sheets and in cholesterol content. This spectral behavior could be partly explained considering a reduction of protein hydration caused by the surrounding lipids^31^,^68^.

Characterization of these properties *in situ*, at the individual level, is unprecedented. The biophysical features of the fibrils may be related to their specific pathogenic profile, including organ tropism and degree of organ dysfunction, and to the histological properties of the amyloid deposits. Assessment of the properties of fibrils by FTIR analysis of a larger number of clinically annotated tissue samples is very likely to provide novel information of translational relevance.

Overall, this work paves the way for the development of clinical-grade, advanced spectroscopic label-free approaches to monitor protein aggregation and disaggregation in samples. Besides being possible new means for objective diagnosis, these approaches could also lead to a better comprehension of the interplay between fibril formation and the *in vivo* environment where the aggregates are formed, helping to fill the gap in the knowledge about the bases of amyloidosis at the individual level.

## Materials and Methods

### Human tissues

Human subcutaneous adipose tissue samples were acquired from two patients at autopsy and from one individual during biopsy examination for diagnostic purposes. Heart tissue samples were acquired from two AL amyloidosis patients at autopsy; in two additional AL patients, tissue was withdrawn from heart explanted during cardiac transplantation for end-stage amyloid cardiomyopathy (Table 1). Amyloid-negative heart tissues to be used as control were also acquired from hearts explanted during cardiac transplantation for end-stage hereditary and valvular cardiomyopathy (Table 1). The study was approved by the ethical committee of Fondazione IRCCS Policlinico San Matteo and was performed in accordance with the Declaration of Helsinki. Informed consent for acquisition, storage and use of biological samples for research purposes was obtained for all patients described in this study. The presence of amyloid deposits in tissue sections was performed by staining with alkaline Congo red and viewing under polarized light. Amyloid typing in each patient was confirmed by immuno-electron microscopy^5^. Tissues were stored at −80 °C until use; for the purpose of this study, frozen sections of each tissue were embedded in OCT matrix (Thermo Scientific), cryosectioned in 5–10 μm thick slices and mounted on BaF_2_ IR transparent windows. OCT was then accurately removed before FTIR measurements.

### Extraction of amyloid fibrils from heart tissue

Amyloid fibrils were extracted from samples of heart obtained post-mortem from a patient affected by AL amyloidosis, as previously described^69^. As negative control, the same procedure was used to process one of the amyloid-negative heart specimens described above. The material was homogenised in 10 ml of 10 mM Tris/EDTA, pH 8.0, containing 1.5 mM phenylmethylsulfonyl fluoride (PhMeSO2F)/100 mg of tissue, and centrifuged at 60,000 g in an ultracentrifuge (Beckman L8-704; Beckman Instruments) for 30 min before the supernatant was removed. This step was repeated five times until the absorbance measurement at 280 nm was less than 0.2; subsequently, the pellet was homogenized in water in the presence of 1.5 mM PhMeSO2F and centrifuged at 60,000 g for 30 min. Eight aqueous fractions were obtained. The yield in fibrils was monitored by microscopic analysis of the extracted material stained with Congo red. Aqueous fractions that were Congo red positive, were lyophilized and suspended in 20 mM sodium phosphate, pH 7.4, before FTIR measurements in attenuated total reflection (ATR)^15^,^28^.

### Recombinant LC production

Recombinant full length LC HT3 was produced in *E. coli*^12^. Nucleotide sequence of selected LC was obtained using a universal inverse-PCR strategy. In order to determine germline gene, nucleotide sequence alignment was made using the current releases of EMBL-GenBank, V-BASE (V BASE Sequence Directory, MRC Centre for Protein Engineering, Cambridge, UK) and IMGT sequence directories. The amyloidogenic cardiotoxic protein HT3 derived from *IGLV1-51* germline gene. Biochemical characterization on the purified recombinant LC indicated that the recombinant protein was arranged as dimers in solution and exhibited the typical properties of immunoglobulin LC^12^.

### Production of amyloid fibrils *in vitro* from purified LC

Amyloid fibrils were obtained *in vitro* from recombinant full-length light chain of patient HT3, as previously described^12^. Briefly, 1 mg/mL native LC samples were prepared in 10 mM TRIS-HCl pH 7.0, filtered (0.22 μm) and incubated at 60 °C rocking at 250 rpm on a Innova 43R shaker (New Brunswick Scientific, Edison, NJ) for up to 72 h. Aliquots (10 μL) were removed at various time points, added to a blank solution of 20 mM Congo red (Fisher Scientific, Loughborough, UK) in 25 mM PBS buffer at pH 7.4 and left incubated for 30 min at room temperature. Fibril formation was monitored by recording the UV spectrum between 400 and 700 nm on an Infinite M200Pro spectrophotometer (Tecan, Mannedorf, Switzerland). Congo red binding was calculated according to Klunk and coworkers^70^. The fibril pellet obtained by sample centrifugation was suspended in 20 mM sodium phosphate, pH 7.4, before FTIR measurements in attenuated total reflection (ATR)^15^,^28^.

### FTIR microspectroscopy analysis of tissues

FTIR absorption spectra of tissue samples were acquired in transmission mode, between 4000 and 800 cm^−1^, by means of a Varian 610-IR infrared microscope coupled to the Varian 670-IR FTIR spectrometer (both from Varian Australia Pty Ltd), equipped with a mercury cadmium telluride (MCT) nitrogen-cooled detector^17^. The variable microscope aperture was adjusted to ~40 μm × 40 μm. Measurements were performed at 2 cm^−1^ spectral resolution; 25 KHz scan speed, triangular apodization, and by the accumulation of 512 scan co-additions.

Absorption spectra are shown as measured, without any baseline correction. Water vapor subtraction was performed when necessary^17^.

Second-derivative spectra^30^ were obtained following the Savitsky-Golay method (third-grade polynomial, 9 smoothing points), after a binomial 13 smoothing points of the measured spectra, using the GRAMS/32 software (Galactic Industries Corporation, USA).

To verify the reproducibility and reliability of the spectral results, 50–100 areas for each sample were measured. In the figures we showed selected spectra representative of the analyzed tissues. The second derivative spectra were reported in the Amide I and II bands after normalization at the tyrosine peak around 1515 cm^−1^, while between 3000–2800 cm^−1^ and 1500–1200 cm^−1^ after normalization at the CH_3_ ~2957 cm^−1^ band.

### ATR-FTIR spectroscopy analysis of recombinant LC and of *ex vivo* fibrils from heart tissue

FTIR absorption spectra of recombinant LC and of *ex vivo* fibrils from heart tissue were acquired by means of a single reflection ATR device (Quest, Specac, USA) coupled to the Varian 670-IR FTIR spectrometer (Varian Australia Pty Ltd). Aliquots of 2 μL of the protein samples were deposed on the diamond crystal of the ATR device and dried at room temperature in order to obtain a protein hydrated film^15^,^28^. FTIR spectra were collected under the following conditions: 2 cm^−1^ spectral resolution, scan speed of 25 kHz, 1000 scan coadditions, triangular apodization, and a nitrogen-cooled Mercury Cadmium Telluride detector. Measured spectra were analyzed as described in the previous section (FTIR microspectroscopy analysis of tissues).

## Additional Information

**How to cite this article**: Ami, D. *et al.* In situ characterization of protein aggregates in human tissues affected by light chain amyloidosis: a FTIR microspectroscopy study. *Sci. Rep.* **6**, 29096; doi: 10.1038/srep29096 (2016).

## Supplementary Material

Supplementary Information

## Figures and Tables

**Table 1:** Main clinical and histological features of the samples analyzed in this study.

Sample code	Autopsy (A)/Biopsy (B)	Tissue type	Patient’s features	
			Disease	Amyloid organ involvement
AT1	B	Adipose tissue	Amyloid negative	n.a.
AT2	A	Adipose tissue	AL λ	Heart
AT3	A	Adipose tissue	AL λ	Kidney, heart
HT1	A	Heart tissue (LV)	AL λ	Heart
HT2	A	Heart tissue (LV)	AL λ	Heart, kidney, liver
HT3	B	Heart tissue (LV)	AL λ	Heart
HT4	B	Heart tissue (LV)	AL λ	Heart
HT5	B	Heart tissue (LV)	Amyloid negative	n.a.
HT6	B	Heart tissue (LV)	Amyloid negative	n.a.

LV: left ventricle; n.a.: not applicable.
