# Supplementary material for: In situ characterization of protein aggregates in human tissues affected by light chain amyloidosis: a FTIR microspectroscopy study
Source: Sci Rep. 2016 Jul 4;6:29096. doi: 10.1038/srep29096 (PMC4931462; doi:10.1038/srep29096)
Supplement: Supplementary Information [file srep29096-s1.pdf]

SUPPLEMENTARY INFORMATION FOR:

In situ characterization of protein aggregates in human tissues affected by light chain amyloidosis: a FTIR microspectroscopy study

Diletta Ami[1,2,*], Francesca Lavatelli[3], Paola Rognoni[3], Giovanni Palladini[3], Sara Raimondi[4], Sofia Giorgetti[4], Luca Monti[4], Silvia Maria Doglia[1,2], Antonino Natalello[1], Giampaolo Merlini[3,*]

[1] Department of Biotechnology and Biosciences, University of Milano-Bicocca, Piazza della Scienza 2, 20126 Milano, Italy

[2] Department of Physics, University of Milano-Bicocca, Piazza della Scienza 3, 20126 Milano, Italy

[3]Amyloidosis Research and Treatment Center, Foundation IRCCS Policlinico San Matteo, and Department of Molecular Medicine, University of Pavia

[4] Department of Molecular Medicine, Institute of Biochemistry, University of Pavia, via Taramelli 3b, 27100 Pavia

*Correspondence and requests for materials should be addressed to D.A. (e-mail: diletta.ami@unimib.it) and G.M. (e-mail: gmerlini@unipv.it)

Figure S1

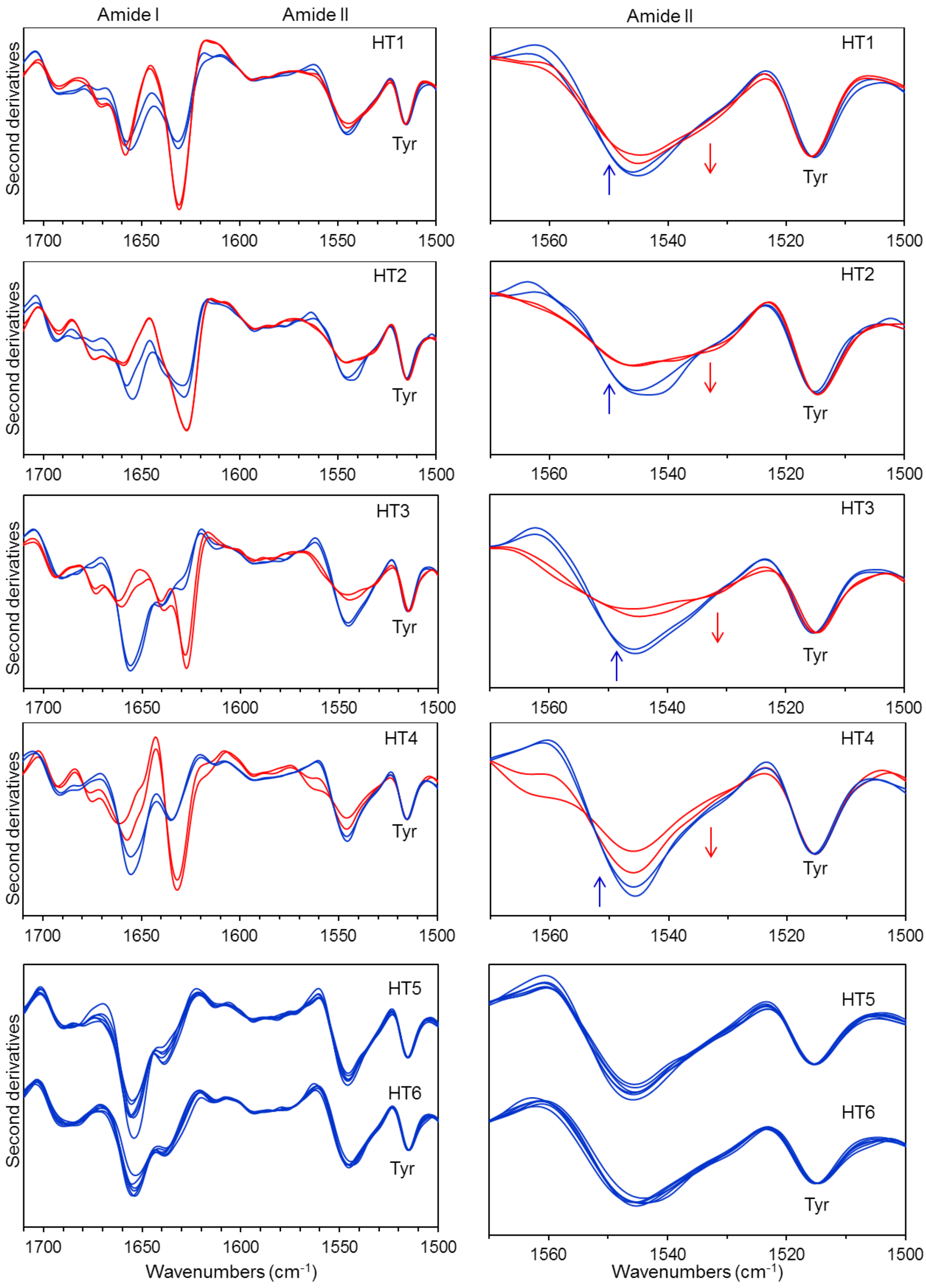

Figure S1. Amide II band of cardiac tissues.

The second derivatives of the FTIR absorption spectra of cardiac tissues are reported between 1700 and 1500 cm^{-1} spectral range (left panels) to appreciate the normalization at the tyrosine peak at ~

1515 cm^{-1}. A magnification of the derivative spectra in the Amide II band (right panels) is reported to better visualize spectral changes in sample areas characterized by a different extent of protein aggregates. In amyloid-positive tissues, the spectra of areas enriched in amyloid deposits are displayed in red.

Figure S2

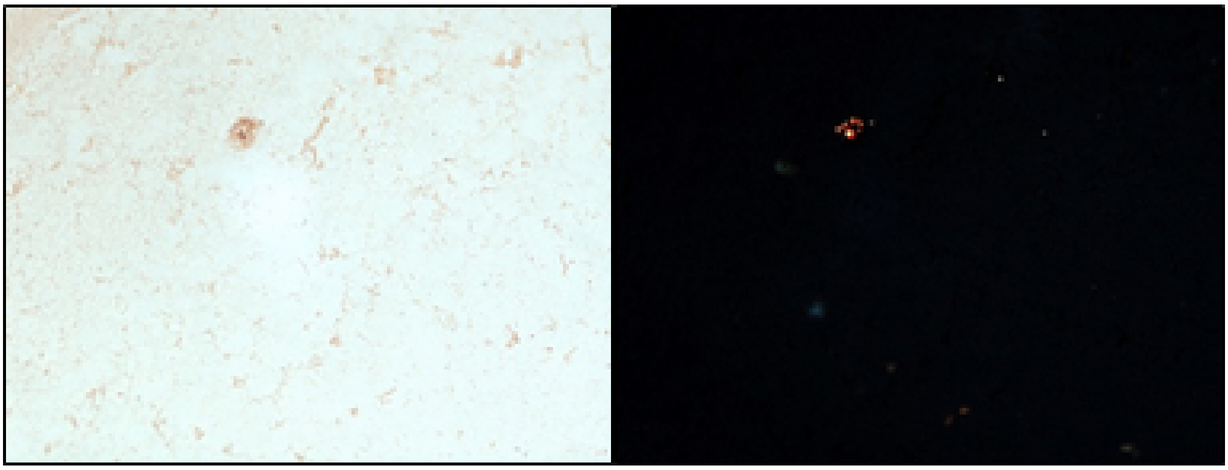

Figure S2. Analysis of the material extracted from the amyloid negative tissue HT6*. Congo Red staining of the water-extracted material visualized under light (left) and polarized (right) microscopy. Magnification 200x.*

Figure S3

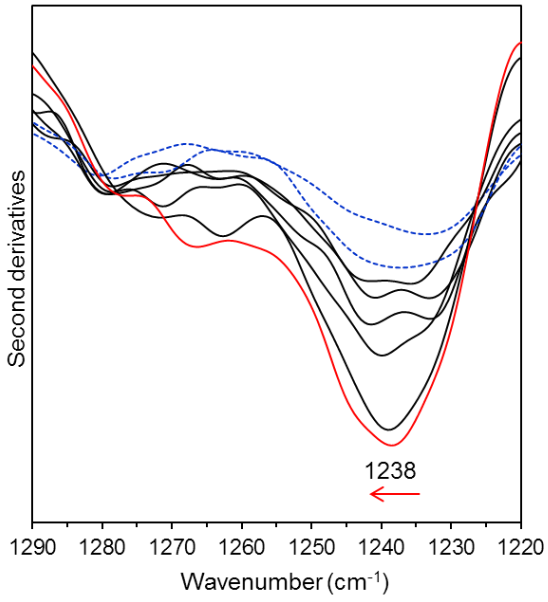

Figure S3. Magnification of the complex IR absorption band due to the overlapping contribution of phosphates, Amide III and GAGs, in HT1 tissue.

We reported the same second derivative spectra of figures 3 and 4. The arrow points to the upshift of the ~ 1233 cm^{-1} phosphate band to 1238 cm^{-1}, induced by the absorption of GAGs and collagen and by the presence of cholesterol.

Figure S4

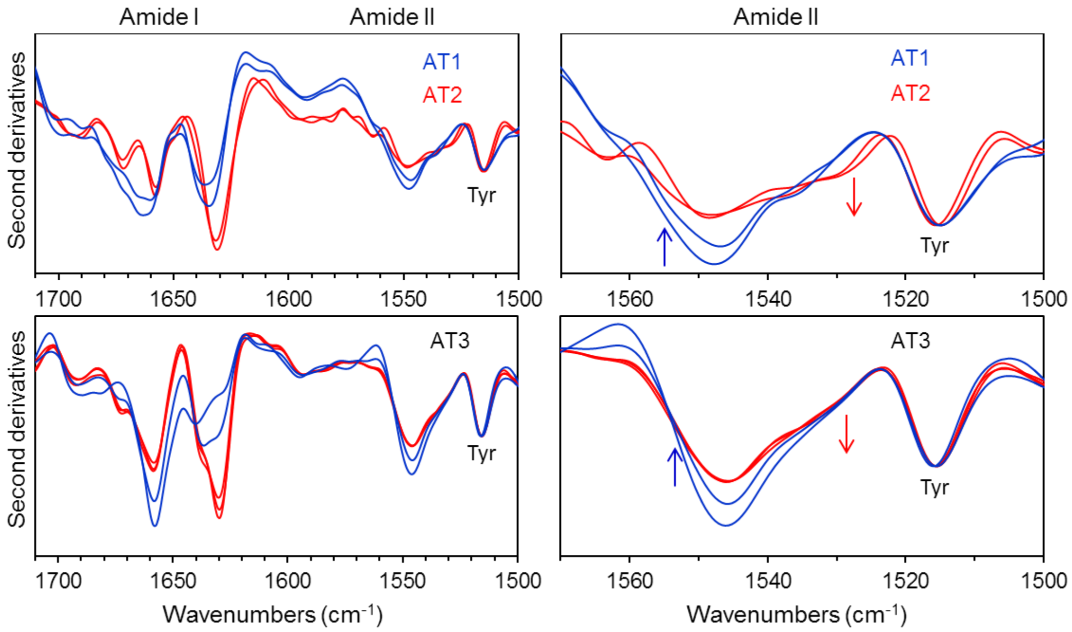

Figure S4. Amide II band of adipose tissues.

The second derivatives of the FTIR absorption spectra of adipose tissues are reported between 1700 and 1500 cm^{-1} spectral range (left panels) to appreciate the normalization at the tyrosine peak at ~ 1515 cm^{-1}. In the upper panel, a comparison between amyloid positive (AT2) and negative (AT1) tissues is shown. For AT3 sample, the spectra of areas enriched in amyloid deposits are displayed in red (bottom panel). A magnification of the derivative spectra in the Amide II band (right panels) is displayed to better visualize spectral changes in sample areas characterized by a different extent of protein aggregates.
